# Coeliac Disease and Microscopic Colitis: The Largest Study Assessing Prognosis and Risk of Hospital Admission

**DOI:** 10.3390/nu16132081

**Published:** 2024-06-29

**Authors:** Suneil A. Raju, Megan E. Rawcliffe, Freya J. Bowker-Howell, Mohamed G. Shiha, Kamaldeep E. Kaur, Jonathan Griffin, Simon S. Cross, David S. Sanders

**Affiliations:** 1 Academic Unit of Gastroenterology, Sheffield Teaching Hospitals National Health Service Foundation Trust, Sheffield S102JF, UKdavid.sanders1@nhs.net (D.S.S.); 2 Division of Clinical Medicine, School of Medicine and Population Health, University of Sheffield, Sheffield S102RX, UK

**Keywords:** microscopic colitis, coeliac disease, hospital admission, hospitalisation

## Abstract

Microscopic colitis (MC) and coeliac disease (CD) are common associated gastrointestinal conditions. We present the largest study assessing hospitalisation in patients with MC and the effect of a concomitant diagnosis of CD. Data were retrospectively collected between January 2007 and December 2021 from all patients diagnosed with MC and compared to a database of patients with only CD. In total, 892 patients with MC (65% female, median age 65 years (IQR: 54–74 years) were identified, with 6.4% admitted to hospital due to a flare of MC. Patients admitted were older (76 vs. 65 years, *p* < 0.001) and presented with diarrhoea (87.7%), abdominal pain (26.3%), and acute kidney injury (17.5%). Treatment was given in 75.9% of patients, including intravenous fluids (39.5%), steroids (20.9%), and loperamide (16.3%). Concomitant CD was diagnosed in 3.3% of patients and diagnosed before MC (57 versus 64 years, *p* < 0.001). Patients with both conditions were diagnosed with CD later than patients with only CD (57 years versus 44 years, *p* < 0.001). In conclusion, older patients are at a higher risk of hospitalisation due to MC, and this is seen in patients with a concomitant diagnosis of CD too. Patients with MC are diagnosed with CD later than those without.

## 1. Introduction

Microscopic colitis (MC) is an inflammatory condition caused by histological changes to the large bowel. The predominant symptoms in MC are watery and severe diarrhoea, faecal incontinence, nocturnal symptoms, and abdominal pain [1]. It is more common in individuals with autoimmune conditions and those on proton pump inhibitors (PPIs) (OR: 2.68, 95% CI1.73–4.17), serotonin-specific receptor inhibitors (SSRIs) (OR: 2.41, 95% CI 1.64–3.53), non-steroidal anti-inflammatories (NSAIDs) (OR: 1.5, 95% CI1.14–1.96), and statins (OR: 1.31, 95% CI1.05–1.62) [1,2]. It is also more common in females than males (OR: 1.92–3.05:1) [2].

MC is divided into lymphocytic colitis (LC), collagenous colitis (CC), and incomplete MC (MCi), which all have an increased inflammatory infiltrate in the lamina propria. However, in LC there is an increased number of intraepithelial lymphocytes (IELs) of ≥20 per 100 surface epithelial cells, and in CC, a thickened subepithelial collagenous band >10 µm [1]. MCi patients have incomplete histological changes of either LC or CC. Often the large colon appears macroscopically normal, and therefore diagnosis is dependent on biopsy assessment [3]. This macroscopically normal appearance of the large bowel increases the risk of an MC diagnosis being missed. Even when a colonoscopy is complete to explicitly rule out microscopic colitis, one study found biopsies were only taken correctly for 48% of patients, and overall, when assessing a patient with diarrhoea, no biopsies were taken in 8.6% of cases [4]. Furthermore, there is downward pressure within many countries to minimize the number of referrals made, further increasing the risk of a missed diagnosis [5]. As a result, patients may be diagnosed with diarrhoea-predominant irritable bowel syndrome (IBS-D). One retrospective study of 247 patients initially diagnosed with IBS-D who subsequently had a colonoscopy found that 6% of patients actually had undiagnosed MC [6]. There is therefore a need for greater recognition of this condition.

If diagnosed, MC has treatment options. The United European Gastroenterology (UEG) and European Microscopic Colitis Group (EMCG) support the use of budesonide 9 mg per day for patients with active MC to induce remission [1]. Treatment results in a significant improvement in quality of life and clinical and histological remission [7,8,9]. However, there are challenges with treating patients with budesonide. A pooled analysis of three studies found that only 68% of patients receiving budesonide maintained remission, and some patients may not wish to be on long-term corticosteroids or are intolerant [8]. There is therefore a need for other treatment options. Bile acid sequestrants (BASs), which have been shown to improve the quality of life of patients with bile acid malabsorption (BAM) and Crohn’s disease, are currently only recommended in patients with MC who also have BAM [1,10,11]. However, a recent study of 282 patients with MC who were treated with bile acid sequestrants (BASs) reported a complete or partial response in 65.6% of patients. Interestingly, of the patients who were tested for BAM, no association was found between response and presence of BAM or previous cholecystectomy [12]. There is therefore a need for more research into treatment options and conditions associated with MC.

Coeliac disease (CD) is a chronic, autoimmune, gluten-sensitive enteropathy that occurs in susceptible individuals. The presenting gastrointestinal symptoms in CD, similar to those in MC, include chronic diarrhoea and abdominal pain; however, malabsorption is also seen in CD [13,14]. A meta-analysis of 26 studies found CD to be significantly associated with MC (OR:8.276, 95% CI 5.888–11.632). The pooled event rate of concomitant MC found in patients with CD was 6.7% (95% CI: 4.4–10.0%), and the pooled event rate of CD found in patients with MC was 7.7% (95%CI: 4.6–12.6%) [15]. The histological changes seen in the large colon of patients with MC are somewhat similar to those seen in the duodenum of patients with coeliac disease (CD), which alludes to a potentially similar pathophysiological response or common aetiology [14,16]. This is further supported by a Finnish study of 80 patients with MC, which (similar to findings in CD) found a higher frequency of the human leukocyte (HLA) haplotype DR3-DQ2 compared to controls without MC (43.8% vs. 18.1, *p* < 0.001) [17].

Both MC and CD are under recognised, and therefore the opportunity to treat these conditions is often missed [5,18,19]. Furthermore, due to their gastrointestinal symptomatic burden and the challenges of treating these conditions, patients experience a lower quality of life [20,21,22]. We therefore wished to examine the clinical importance of these conditions, and whilst the association is well recognised, the clinical significance of concomitant CD in patients with MC is unknown. We present the largest study assessing the risk of hospitalisation due to MC and assess the role of CD in these patients.

## 2. Methods

All patients diagnosed with histologically confirmed MC at Sheffield Teaching Hospitals National Health Service (NHS) Foundation Trust, United Kingdom, between January 2007 and December 2021 were identified. Their data were retrospectively collected from case notes and endoscopy reports with regards to their symptoms, histology of gastrointestinal biopsies, serology, and medical history.

### 2.1. Patients

MC was diagnosed based on histological findings on colonic biopsies as per the United European Gastroenterology and European Microscopic Colitis Group statement and recommendations as described above [1]. Based on these findings, patients were divided into LC, CC, and MCi. All histology samples were reported by expert histopathologists with an interest in gastroenterology. CD was diagnosed based on villous atrophy on duodenal biopsies in the presence of positive immunoglobulin A (IgA) tissue transglutaminase (tTG) or anti-endomysial antibody (EMA). Investigation for CD was only completed when clinically indicated.

Patients with both MC and CD were then age and sex matched to patients with only CD. Patients with CD were identified from The Sheffield United Kingdom Coeliac Research Database. The serology and genotype of these patients were then compared. A further analysis of the age at diagnosis was made comparing patients with both MC and CD to those with only CD from the same database.

### 2.2. Statistics

All clinical data were anonymised prior to analysis. Patients underwent clinical tests and assessments as part of their routine clinical care. Data handling was completed using Microsoft Excel (2016) and analysis in IBM SPSS V27.0 (IBM). Associations between dichotomous variables, such as admission to hospital, were calculated using chi-squared test, and normally distributed continuous variables were analysed using the Student’s *t*-test.

## 3. Results

### 3.1. Characteristics of Patients with Microscopic Colitis

In total, there were 892 patients with MC (65.0% female, median age 66 years (IQR: 55–75 years)). The majority had lymphocytic colitis, followed by collagenous colitis, indeterminate microscopic colitis, and giant cell colitis (69.8%, 25.8%, 4.1%, and 0.2% respectively). There was no difference in the gender (*p* = 0.128) or age (*p* = 0.209) of patients based on whether they were diagnosed with LC or CC.

The most common presenting symptoms were diarrhoea, abdominal pain, and weight loss (Table 1). In total, 3.3% had a diagnosis of CD, and 51.0% were on either a PPI, SSRI, beta blocker, non-steroidal anti-inflammatory, or statin (Table 2).

### 3.2. Hospitalisation in Patients with Microscopic Colitis

In total, 6.4% of patients were admitted due to a flare of their MC (median length of hospital admission: 10 days; IQR: 4–20 days), but a prior diagnosis had only been made in 7% of cases. Of these patients, 22.8% had had at least one previous colonoscopy for diarrhoea or rectal bleeding (median number of previous colonoscopies: 2 (IQR:1–2)). Patients admitted were older (median 76 (IQR:61–81) years versus 65 (IQR: 54–74) years (*p* < 0.001)). Of those admitted, 87.7% presented with diarrhoea, 26.3% with abdominal pain, 17.5% with acute kidney injury (AKI), 17.5% with rectal bleeding, 16.4 with vomiting, and 7.0% following a collapse or loss of consciousness. Of these patients 75.9% had treatments recorded, including starting intravenous (IV) fluids (39.5%), starting steroids (20.9%), starting loperamide (16.3%), and other treatments (18.6%), including stopping their PPI or SSRI or starting creon, buscopan, antiemetics, or mesalazine. A further 0.8% of patients were admitted for infection, shortness of breath, fractures, or neurological symptoms and subsequently developed a flare of their MC during their admission (median length of hospital admission: 51 days; IQR: 28–112 days).

### 3.3. Characteristics of Patients with Microscopic Colitis and Concomitant Coeliac Disease Diagnosis

There were 29 patients with MC who were also diagnosed with coeliac disease (72.4% female; median age at time of CD diagnosis: 57 years; IQR: 44–67 years). Of those, 5 patients had collagenous colitis and 24 had lymphocytic colitis. Patients with MC and concomitant CD had a similar presentation (Table 1). There was no difference in the number of symptoms patients with MC had versus those with MC and a concomitant diagnosis of CD (*p* = 0.22). The diagnosis of MC was later than CD in these patients (64 years (IQR:57–71) versus 57 years (IQR:44–67), *p* < 0.001). There was no difference in IgA-tTG (*p* = 0.950), IgA-EMA (*p* = 0.217), or HLA type (*p* = 0.329) for patients with both MC and CD compared to those with CD alone. There was no difference in admission rates between patients with MC and CD versus MC alone (*p* = 0.158).

Patients with MC and concomitant CD were then compared to 2072 patients with CD alone (68.4% female; median age: 44 years (IQR:30–58 years). Patients with MC were diagnosed with CD later than in patients with only CD (57 years (IQR: 44–67) versus 44 years (IQR: 30–58), *p* < 0.001).

## 4. Discussion

To date, this is the largest study to assess hospitalisation in patients with MC and assess the effect of a concomitant diagnosis of CD. In total, 7.2% of patients were hospitalised due to a flare of MC or experienced this during their hospital admission. The majority did not have their MC diagnosed prior to admission. Patients admitted were older and required treatment primarily with IV fluids, steroids, and loperamide. Patients with CD were biochemically indistinguishable from patients with MC and concomitant CD. They were also diagnosed with MC after their diagnosis of CD. CD was not associated with hospitalisation.

### 4.1. Hospitalisation in Patients with Microscopic Colitis

There has only been one study (in a French cohort) of 130 patients with MC assessing the rate of hospitalisation [23]. In this retrospective study, 15% of patients were hospitalised for a similar length of time to our study. The length of admission and presentation of symptoms were the same in our study; however, we have also identified the age at diagnosis to be greater in patients who are hospitalised due to a flare of their MC. Furthermore, we have also identified patients who were admitted for other reasons and subsequently developed a flare of MC during their admission. It is therefore important to consider the diagnosis of MC in hospitalised patients presenting with common gastrointestinal symptoms.

The length of stay of patients who developed MC after being admitted to hospital for another reason was longer. The cost of a general ward bed per day in the NHS is £587 [24]. Therefore, a hospital stay of 10 days would cost £5870 and a stay of 51 days would cost £29,937. The impact of a flare of MC is therefore considerable on the health service as well as on patients. It is difficult to establish means to reduce the length of admission with the current data; however, only 7% of patients were diagnosed with MC prior to their admission, although 22.8% had had a previous colonoscopy for symptoms suggestive of MC. If the diagnosis was made previously, then treatment could have been started in the outpatient setting or rescue therapy with budesonide or loperamide could have been offered. This may have reduced the need for admission. The benefit of this would need further research and careful consideration to prevent other diagnoses being missed in these older patients who present with diarrhoea.

Whilst MC is regarded as a cause of non-bloody diarrhoea, 17.5% presented with rectal bleeding. Previous reports of per rectal bleeding have also been described though not commented on [25]. It is difficult to establish the cause of this in our study, but it may be due to other causes of bleeding, such as haemorrhoids or anal fissures secondary to diarrhoea [25].

### 4.2. Concomitant Coeliac Disease in Patients with Microscopic Colitis

We did not demonstrate an association between patients diagnosed with both MC and CD and admission to hospital due to a flare of MC. However, similar to previous studies, we have shown that the prevalence of CD is higher in patients with MC compared to the general population [26]. In our study, 3.3% of patients with MC had CD, whilst in the general population it is 1% [27]. MC is a potentially undiagnosed condition due to the overlap with irritable bowel syndrome, pressure to reduce the number of colonoscopies, biopsies not being taken, and a lack of biomarkers [5,28,29]. Given the greater prevalence of MC in patients with CD, it is important that all patients with persisting symptoms in CD, particularly if following a gluten-free diet (GFD), should be tested for MC [14].

We have shown that patients are diagnosed with CD before MC by on average seven years. The association between MC and CD may be due to a common aetiology, as both conditions have intraepithelial lymphocytosis, an increased likelihood of human leukocyte antigen-DQ2, and a T-helper-mediated response [14,30,31,32]. However, no association has been demonstrated between gluten intake and the risk of MC [33]. Although the significance of the association is unclear, the importance of screening for MC when CD is present and vice versa is recommended [1,14].

### 4.3. Limitations

There are limitations to this study. Firstly, there is the inherent weakness of its retrospective design; however, patient data were cross-referenced between case notes and endoscopy reports to assess for reliability. Secondly, admissions to hospital were only checked at Sheffield Teaching Hospitals NHS Foundation Trust and therefore may be underestimated. Thirdly, the rate of concomitant CD may be underestimated, as all patients with MC did not undergo serological screening to assess for CD; however, an association has still been demonstrated.

## 5. Conclusions

In conclusion, this is the largest study to report hospitalisation due to a flare of MC. Almost a quarter of patients with MC have had a previous colonoscopy for similar symptoms, representing a potentially missed opportunity to diagnose MC. The prevalence of CD in patients with MC is higher than the general population, and therefore screening for CD is important, particularly in older patients. The later diagnosis of MC in patients with CD merits further investigation. It is hoped that in recognising the risk of hospitalisation in these patients, investigations and treatment can be offered to reduce the burden of hospital admissions and improve patient care.

## Figures and Tables

**Table 1:** Presenting symptoms of patients with microscopic colitis.

Symptom	All % (*n* = 893)	Collagenous Colitis % (*n* = 231)	Lymphocytic Colitis % (*n* = 623)	Indeterminate Microscopic Colitis % (*n* = 37)	Giant Cell Colitis % (*n* = 2)	Microscopic Colitis and Concomitant Coeliac Disease % (*n* = 29)
Diarrhoea	89.3	91.3	88.1	94.6	100	89.7
Abdominal pain	36.6	39.6	35.7	32.4	50	41.4
Urgency	22.0	18.4	23.2	24.3	0	34.5
Weight loss	21.6	19.6	22.7	16.2	0	13.8
Rectal bleeding	20.3	20.4	20.6	16.2	0	20.7
Bloating	13.4	11.0	14.2	15.2	0	17.2
Nocturnal bowel habits	12.5	15.0	11.6	11.4	0	10.3
Faecal incontinence	11.7	12.9	11.2	14.7	0	24.1
Flatulence	8.3	6.7	9.2	3	0	10.3
Mucus in stool	6.8	7.1	6.9	3	0	10.3
Nausea	4.1	4.3	4.2	0	0	13.8
Fatigue	4	3.0	4.3	6.1	0	6.9
Indigestion	2.9	1.4	3.7	0	0	3.4

**Table 2:** Percentage of patients on medications associated with microscopic colitis.

Medication	%
Proton pump inhibitors	31.6
Statin	24.5
Beta blockers	14.3
Angiotensin-converting enzyme inhibitors	12.5
Serotonin-specific receptor inhibitors	13.8
Aspirin	11.5
Non-steroidal anti-inflammatory drugs	9.6

## Data Availability

The dataset is available on request from the authors.

## Abbreviations

AKI	acute kidney injury
BAM	bile acid malabsorption
BAS	bile acid sequestrants
CC	collagenous colitis
CD	coeliac disease
CI	confidence interval
EMA	anti-endomysial antibody
GFD	gluten-free diet
HLA	human leukocyte antigen
IEL	intraepithelial lymphocytes
IgA	immunoglobulin A
IQR	interquartile range
IV	intravenous
LC	lymphocytic colitis
MC	microscopic colitis
MCi	incomplete microscopic colitis
NHS	National Health Service
NSAIDs	non-steroidal anti-inflammatories
OR	odds ratio
PPI	proton pump inhibitor
SSRI	serotonin-specific receptor inhibitor
tTG	tissue transglutaminase
UK	United Kingdom
